# Adaptation and Vulnerability in Chronic Pain: A Study of Profiles Based on Clinical and Psychological Factors

**DOI:** 10.3390/ejihpe15090168

**Published:** 2025-08-23

**Authors:** Juan José Mora-Ascó, Carmen Moret-Tatay, María José Jorques-Infante, María José Beneyto-Arrojo

**Affiliations:** 1 Escuela de Doctorado, Universidad Católica de Valencia “San Vicente Mártir”, 46001 Valencia, Spain; 2 Facultad de Psicología, Universidad Católica de Valencia “San Vicente Mártir”, 46100 Valencia, Spain; mj.jorques@ucv.es (M.J.J.-I.); mariajose.beneyto@ucv.es (M.J.B.-A.)

**Keywords:** chronic pain, coping profiles, psychological factors

## Abstract

Introduction. Chronic pain (CP) is a multidimensional condition that exerts a considerable impact on individuals’ quality of life and presents a wide range of clinical and psychological expressions. This study sought, firstly, to identify distinct clinical profiles among individuals with CP based on clinical indicators, and secondly, to examine the differences in psychological vulnerability and pain-related coping strategies according to the clinical profiles. Methods. A total of 251 adults diagnosed with CP and residing in Spain participated in the study. Participants completed the Purpose in Life Test, the Reflective Functioning Questionnaire, the Interpersonal Needs Questionnaire, the Beck Hopelessness Scale, the Difficulties in Emotion Regulation Scale, and the Pain Coping Questionnaire. A two-step cluster analysis was performed to identify subgroups within the sample, followed by independent samples *t*-tests to assess psychological differences between clusters. Results. This study identified two clinical profiles among individuals with CP, distinguished by diagnostic delay, disease progression, and functional impact. Cluster 1 exhibited greater functional impairment, lower quality of life, and higher emotional distress (uncertainty, perceived burdensomeness, emotional dysregulation, and hopelessness). In contrast, Cluster 2 showed lower functional impairment, better quality of life, greater use of distraction strategies, and a higher meaning in life. Discussion. These findings suggest that both medical and psychological aspects appear to be associated with each other and may influence the perception, evolution and adaptation to CP.

## 1. Introduction

Chronic pain (CP) poses a significant challenge to global public health (Langley et al., 2011), affecting an estimated 30% of the world’s population (Cohen et al., 2021) and approximately 25.9% of individuals in Spain (Dueñas et al., 2024). Clinically, this condition is defined as a pain experience that persists for more than three months and continues even after the resolution of the original injury or illness (Treede et al., 2019). Far from being confined to a strictly physical dimension, this emerges from the complex interplay of biological, psychological, and social factors (Edwards et al., 2016), producing a progressive deterioration in the Quality of Life (QoL) of those affected (Langley et al., 2011).

In this context, it becomes essential to combine the clinical and psychological variables that mark the onset and evolution of pain. In this regard, Turk and Rudy (1988) highlighted the importance of identifying variables that influence the development and maintenance of chronic illness by integrating physical, psychological, and social dimensions. Based on this approach, they empirically established three patient clusters. The first group was characterized by more severe disease progression, significant limitations in daily functioning, and marked psychological distress. The second cluster comprised individuals experiencing elevated interpersonal distress and reporting low levels of perceived family and social support. The third group showed lower pain intensity, greater emotional stability, and minimal disruption in QoL.

Expanding this theoretical approach, among the clinical factors explored in the available literature, the duration of the condition, diagnostic delay, and the level of interference in QoL have been identified as key elements that not only determine the functional impact of the illness but also influence how individuals respond to their symptoms (Burke et al., 2020; Lynch et al., 2008). In fact, prolonged delays in receiving an accurate diagnosis have been consistently associated with increases in variables related to psychological distress, such as emotional dysregulation, heightened levels of hopelessness, the adoption of less adaptive coping responses, and a diminished Meaning in Life (MiL) (Liddy et al., 2017; Semple & Hogg, 2012). These factors seem to affect how individuals personally experience the CP (Erfani et al., 2015).

Among these, hopelessness emerges as a particularly detrimental cognitive factor that can intensify pain perception and, over time, reduce the motivation to engage in reinforcing or meaningful activities (Wang et al., 2020). In this sense, recent studies have highlighted the association between hopelessness and affective disturbances, such as anxiety and depressive symptoms, which further hinder the development of effective coping strategies (Lerman et al., 2015; Solomon et al., 2024).

In a distinct yet equally relevant domain, emotion regulation stands out as a core psychological component. It refers to the individual’s ability to become aware of internal affective states, set regulation goals, and implement appropriate abilities to manage emotional responses (McRae & Gross, 2020). When these skills are underdeveloped, the individual’s perception of pain may intensify—particularly under conditions of elevated stress that interfere with the patient’s capacity to cope effectively with the condition (Norman-Nott et al., 2024).

Beyond these mechanisms, perceived burdensomeness (PB) has emerged as a significant indicator of psychosocial distress in individuals with CP. Existing literature points to the progressive course and worsening of the condition—especially in the absence of adequate treatment, as key contributors to emotional, relational, and functional overload (Wilson et al., 2017). As such, the subjective sense of being a burden may arise from feelings of helplessness, frustration related to symptom control, and uncertainty about the future (Kowal et al., 2012).

In contrast, MiL appears as a protective psychological resource throughout the CP process. Thus, having a meaningful life purpose has been consistently linked to more adaptive responses to illness and to the development of resilient attitudes (Miao & Cao, 2024). In this sense, the evidence suggests that patients who are able to reframe their illness-related adversity often report higher levels of QoL and a reduced perception of pain intensity, underscoring the pivotal role that this existential factor may play they are in coping (Reed et al., 2024).

In parallel, reflective functioning, understood as the ability to recognize and interpret both one’s own mental states and those of others, also plays a positive role in the management of CP. This capacity enables individuals to make sense of their bodily experiences and come to terms with the physical consequences of their condition, which, in turn, can facilitate more adaptive coping and reduce maladaptive responses to suffering (Holzer et al., 2024).

Coping strategies, likewise, are key elements in the psychological adjustment to this situation. The use of active, intentional management mechanisms, such as conscious distraction, has been consistently associated with enhanced QoL and improved psychological well-being in individuals with persistent pain. In contrast, reliance on avoidant or passive strategies tends to reinforce both emotional distress and functional limitations (Kristofferzon et al., 2018; Pereira et al., 2020).

Taken together, the interaction between clinical and psychological variables contributes to a more nuanced understanding of the heterogeneity in patients’ adaptive profiles. These dimensions appear to be interconnected in a bidirectional manner. On the one hand, medical factors may exert a primary influence, which can be either exacerbated or alleviated by psychological processes (Kak et al., 2025). On the other hand, shifts in illness perception and mood may alter how the individual experiences impairment and disability (Molton et al., 2009). In this regard, variables such as disease duration, time to diagnosis, and interference with QoL not only describe the physical condition but also shape the psychological landscape in which vulnerability or resilience to CP develops (Fillingim, 2017; Hansen et al., 2015).

Based on this association of group variables, the present study aimed to (1) to identify distinct clinical profiles among individuals with CP based on clinical indicators; and (2) to examine the differences in psychological vulnerability and pain-related coping strategies according to the clinical profiles. Addressing these aspects may contribute to a better understanding of adaptation trajectories in people living with CP, suggesting a basis for identifying risk and protective factors that influence patients’ coping and QoL.

## 2. Materials and Methods

### 2.1. Participants

The sample for this study consisted of 251 adult participants residing in various autonomous communities across Spain. The majority were from the Valencian Community (38.6%) and the Community of Madrid (37.5%). Other represented regions included Castilla-La Mancha (6%), the Region of Murcia (4.8%), Andalusia (4.4%), Catalonia (3.2%), Castilla y León (3.2%), and Galicia (2%). Additional participants were drawn from Aragón, Extremadura, the Balearic Islands, Navarre, the Basque Country, and the autonomous city of Melilla, each accounting for less than 1% of the total sample.

Participants’ ages ranged from 18 to 84 years, with a mean age of 50.51 years (SD = 9.93). In terms of sex distribution, a substantial female predominance was observed, 84.5% (n = 212) identified as women, while 15.5% (n = 39) identified as men. Regarding socioeconomic status, 78.9% reported a medium income level, and 33.9% indicated being the primary economic provider within their household. Recruitment was conducted through hospital-based pain management units and patient associations. All participants had received a clinical diagnosis of CP and reported pain intensity levels exceeding 7 on the Visual analog Scale (VAS), which ranges from 1 (minimal pain) to 10 (the most severe pain).

### 2.2. Procedures

Ethical approval for this study was granted by the Ethics Committee of the Catholic University of Valencia “San Vicente Mártir” (reference code: UCV/2022-2023/034/v2). Following this approval, the research team contacted the legal representatives of relevant healthcare institutions and patient organizations to request formal authorization. Five entities ultimately agreed to participate: three patient associations dedicated to CP and two hospital centers with specialized units in pain management, serving individuals from different regions of Spain.

Once institutional authorization was secured, detailed information regarding the study’s aims and procedures was provided to potential participants through direct communication. Informed consent for voluntary participation was then obtained. Recruitment took place between November 2023 and February 2024. During this period, members of the research team visited each participating center to support the data collection process.

Participants were asked to complete a self-administered online questionnaire that assessed sociodemographic and psychological variables. The survey was delivered via Microsoft Forms and completed using participants’ personal mobile devices. All responses were collected and anonymized for statistical analysis.

### 2.3. Materials

Clinical Variables

Following the collection of sociodemographic data (e.g., sex, chronological age) and clinical history related to CP, specifically, the number of years from symptom onset to formal diagnosis (Diagnostic Delay), the duration of the condition since diagnosis (Disease Progression), and the degree of interference and QoL, participants completed the following standardized assessment tools.

Purpose in Life Test (PIL-10; García-Alandete et al., 2013)

The PIL-10 is a self-report instrument designed to assess MIL across two core components: a cognitive-evaluative dimension and a motivational dimension reflecting the individual’s engagement with future-oriented goals and aspirations. The questionnaire includes 10 items, each rated on a 7-point Likert scale, and yields both subscale and total scores.

The factorial structure of the Spanish adaptation was validated by García-Alandete et al. (2013), who confirmed its psychometric soundness in a Spanish-speaking population. The original validation study reported a Cronbach’s alpha of 0.83. In the present sample, the PIL-10 demonstrated excellent internal consistency, with a total scale reliability of α = 0.92.

Reflective Functioning Questionnaire (RFQ-8; Fonagy et al., 2016)

The RFQ-8 is a brief self-report tool designed to assess reflective functioning, or mentalizing capacity, defined as the ability to interpret one’s own and others’ behaviors in terms of underlying mental states. The instrument consists of 8 items measured on a 7-point Likert scale, evaluating two core dimensions: Certainty (RFQ8-C) and Uncertainty (RFQ8-U) regarding mental states.

This instrument was developed by Fonagy and colleagues (2016) based on the original 54-item RFQ. The RFQ-8 has been validated in both clinical and non-clinical Spanish populations and has demonstrated adequate internal consistency and construct validity (Ruiz-Parra et al., 2023). For the present study, the internal reliability coefficients were acceptable, with α = 0.75 for the Certainty subscale and α = 0.77 for the Uncertainty subscale.

Interpersonal Needs Questionnaire (INQ-15; Van Orden et al., 2012)

The INQ-15 is a brief self-report tool composed of 15 items designed to measure key constructs of the Interpersonal Theory of Suicide (Joiner et al., 2012). Specifically, it evaluates perceived burdensomeness (items 1–6) and thwarted belongingness (items 7–15), both considered proximal risk factors for suicidal thoughts and behaviors.

The original 25-item version (Van Orden et al., 2012) was subsequently reduced to enhance its psychometric robustness and facilitate use in research settings. The shortened 15-item format, which was employed in the present study, is widely used due to its brevity and consistent reliability across studies.

Internal consistency in our sample was acceptable, with Cronbach’s alpha values of 0.79 for the burdensomeness subscale and 0.75 for the belongingness dimension, consistent with previous findings reported by Glaesmer et al. (2013).

Beck Hopelessness Scale (BHS; Beck et al., 1974)

The Beck Hopelessness Scale (BHS) is a self-report instrument developed to evaluate negative expectations regarding the future, understood as a core cognitive risk factor for suicidal ideation and behavior. It comprises 20 dichotomous items (True/False) that assess three interrelated domains: feelings about the future, loss of motivation, and negative expectations concerning change or improvement. The total score reflects the degree of hopelessness perceived by the respondent, with higher scores indicating more pronounced cognitive vulnerability.

Originally developed by Beck et al. (1974), the BHS has demonstrated strong psychometric properties across both clinical and non-clinical populations. In the present study, we used the Spanish version translated and adapted by Sánchez-Teruel et al. (2020), which has shown excellent internal consistency and factorial validity. Reliability in our sample was adequate (Cronbach’s α = 0.89), supporting its use as a reliable measure of hopelessness in populations with chronic health conditions.

Difficulties in Emotion Regulation Scale (DERS; Gratz & Roemer, 2004)

The DERS is a self-administered questionnaire developed to evaluate individual difficulties in emotional regulation across multiple domains. It comprises 36 items rated on a 5-point Likert scale, which range from 1 (“almost never”) to 5 (“almost always”), and generates a total score alongside six subscale scores. These subscales assess specific facets of emotional dysregulation, including difficulties in accepting emotional responses, maintaining goal-directed behavior under emotional stress, impulse control problems, limited emotional awareness, perceived lack of access to effective regulation strategies, and poor emotional clarity.

The original version was developed by Gratz and Roemer (2004) and has been widely used in clinical and research contexts due to its strong psychometric properties. In Spain, it was validated by Hervás and Jódar (2008), who reported excellent reliability and factorial validity across both general and clinical populations (α = 0.93). In the present study, internal consistency for the global scale was high (α = 0.91), supporting its utility for assessing regulatory functioning in populations with persistent pain and emotional vulnerability.

Pain Coping Questionnaire—Revised (PCQ-R; Soriano & Monsalve, 2017)

The PCQ-R is a self-report instrument designed to evaluate the coping strategies employed by individuals in response to CP. This version includes 21 items distributed across five subscales that reflect different cognitive-behavioral strategies: self-instructions, distraction, reappraisal, suppression of pain-related thoughts, and active coping. Each item is rated on a Likert scale ranging from 0 (“never”) to 6 (“always”), with higher scores indicating greater frequency of use for each respective strategy.

The scale was developed and validated in Spain by Soriano and Monsalve (2017), based on earlier models of pain coping assessment, and has been extensively used in clinical populations with chronic musculoskeletal or neuropathic pain. The factorial structure of the PCQ-R has been supported by confirmatory factor analyses, and the instrument has shown robust psychometric properties across various samples. Internal consistency for the subscales ranges from α = 0.72 to α = 0.95, indicating adequate to excellent reliability across the different dimensions assessed. In the present study, the reliability indices were acceptable, with internal consistency exceeding α = 0.70 across all subscales and reaching α = 0.85 for the Distraction subscale.

### 2.4. Data Analysis

Descriptive statistics were computed to characterize the sample in terms of sociodemographic, clinical, and psychological variables. A two-step cluster analysis was conducted using years from pain onset to diagnosis, total years of disease progression, and the degree of impairment in basic daily activities as input variables. A K-means cluster analysis was conducted using three variables under study (Diagnostic Delay, Disease Progression and QoL) to identify naturally occurring subgroups within the sample. The optimal number of clusters was determined based on explained variance (R^2^) and silhouette coefficients. Subsequent between-group comparisons were performed using independent samples *t*-tests to examine differences in psychological constructs such as purpose in life, emotional dysregulation, hopelessness, interpersonal needs, and coping strategies. Effect sizes were calculated using Cohen’s d, and significance was evaluated at the *p* < 0.05 threshold. All analyses were conducted using Jasp 0.18.3.

## 3. Results

**Figure 1:**
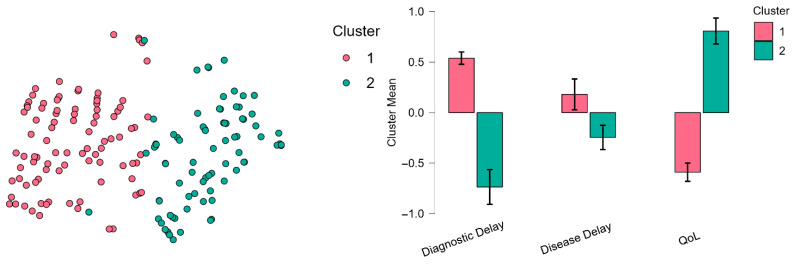
t-SNE Cluster Plot (**Left**) and Cluster Mean Plots (**Right**).

A K-means clustering was conducted using three variables as depicted in Figure 1: the number of years from symptom onset to formal diagnosis (Diagnostic Delay), the duration of the condition since diagnosis (Disease Progression), and the degree of interference with basic daily functioning (QoL).

The optimal two-cluster solution demonstrated moderate clustering performance with an R^2^ of 0.307, indicating that approximately 31% of the variance in the data was explained by the cluster structure. The silhouette coefficient of 0.320 suggested reasonable cluster separation and cohesion.

The analysis revealed two distinct clusters with unequal sizes: Cluster 1 comprised 145 participants (57.8%) while Cluster 2 contained 106 participants (42.2%). The clusters showed differential patterns across three key variables: daily functioning impact indicators, time from pain onset to diagnosis (in years), and current QoL ratings (1–10 scale). Cluster 1 was characterized by participants with higher levels of daily functioning impact (center = 0.538), slightly longer diagnostic delays (center = 0.180), but notably lower QoL scores (center = −0.590). This cluster explained 52.3% of within-cluster heterogeneity and demonstrated a higher silhouette score of 0.370, indicating better internal cohesion. In contrast, Cluster 2 exhibited lower daily functioning impact (center = −0.736), shorter diagnostic delays (center = −0.246), but significantly higher QoL ratings (center = 0.807). This cluster accounted for 47.7% of within-cluster heterogeneity with a silhouette score of 0.243 (Figure 1). To examine differences between clusters in psychological and clinical variables, independent samples *t*-tests were conducted. Table 1 depicts means across cluster groups.

The independent samples *t*-test revealed a significant difference between groups for Sense of life, being higher in the first group, and coping distraction, being higher in the second group (see Table 2).

Cluster 1 also exhibited significantly elevated psychological distress, including higher perceived burdensomeness (M = 21.04 vs. 13.35, Cohen’s d = 0.77), hopelessness (M = 10.64 vs. 6.93, Cohen’s d = 0.71), and emotion dysregulation (M = 86.65 vs. 72.79, Cohen’s d = 0.66). Additionally, Cluster 1 showed greater reflective uncertainty (Cohen’s d = 0.40) and increased use of mental control (Cohen’s d = 0.31) and information-seeking coping strategies (Cohen’s d = 0.45). No significant differences emerged for belongingness, prayer, catharsis, distraction, or self-affirmation measures. These findings indicate that Cluster 1 represents a more psychologically distressed profile characterized by reduced life meaning, elevated emotional difficulties and vulnerability compared to the more resilient Cluster 2.

## 4. Discussion

The present study builds upon previous research that highlights the influence of medical variables related to diagnosis and disease progression in shaping how individuals cope with CP and its psychological impact. In line with these findings, two specific objectives were proposed: (1) to identify distinct clinical profiles among individuals with CP based on clinical indicators; and (2) to examine the differences in psychological vulnerability and pain-related coping strategies according to the clinical profiles.

With respect to the first objective, the two-step cluster analysis revealed the existence of two differentiated clinical profiles among patients with CP. These profiles were established based on three key variables: the number of years since disease onset, the delay between symptom emergence and diagnosis, and the level of impairment in ADL.

The findings described above are consistent with previous research emphasizing the heterogeneity among individuals living with CP (Sil et al., 2021). In this regard, Scheidegger et al. (2023) proposed that patients with CP exhibit substantial variability in disease progression, severity of functional impairment, and associated psychological patterns. Furthermore, existing literature identifies prolonged diagnostic delay as a significant factor contributing to the chronicity of pain and exacerbating its functional consequences (Linton & Shaw, 2011).

In this study, findings indicate that patients grouped in Cluster 2 experienced a shorter course of illness, faced less diagnostic delay, and reported a slightly lower level of functional impairment. This clinical pattern aligns with the notion that early identification and timely medical response may help mitigate the deterioration typically associated with chronic conditions. Prior research supports this view, suggesting that prompt diagnosis is linked to better preservation of daily functioning and a lower likelihood of long-term disability (Lötsch et al., 2019).

In contrast, Cluster 1 encompassed individuals with significantly longer disease duration, a more prolonged wait for diagnosis, and greater interference in QoL. This profile reflects a more severe trajectory, which may place patients at higher risk of entering a cycle of progressive decline. As Breivik et al. (2013) point out, the cumulative burden of CP exerts a tangible toll not only on physical capabilities but also on emotional well-being.

In a similar vein, the relationship between diagnostic delay and the chronicity of pain has been well documented in prior literature. Studies by Tamburin et al. (2014) and Dansie and Turk (2013) suggest that delays in clinical intervention not only exacerbate the pain experience, but also reinforce maladaptive coping patterns, increase disability, and promote central nervous system sensitization to pain. Likewise, the degree of interference in activities in daily life stands out as a critical factor, as high levels of functional disruption have been associated with increased hopelessness and the persistence of disability over time (Reid et al., 2015).

From this perspective, and in light of the previous discussion, the profiles identified in the present study appear to reflect divergent trajectories of adaptation to CP, shaped to a large extent by the duration of unresolved illness and the level of functional impact experienced. Thus, Teixeira et al. (2023) argue that differences in pain duration and its functional interference may significantly modulate both psychological resilience and the capacity for adaptive coping.

The identification of these clinical clusters, based on objective parameters, underscores the importance of addressing medical variables early in the course of persistence pain. This approach aims to support the early detection of at-risk patient profiles. Supporting this rationale, Kamper et al. (2015) emphasize that early interventions targeting patients with a high clinical risk can significantly improve pain outcomes and reduce its long-term psychological and functional impact.

Regarding the second objective, the findings indicate that the clinical variables analyzed, namely, the number of years since disease onset, diagnostic delay, and QoL, exert a significant influence on several indicators of psychological vulnerability in patients living with long pain disease.

To begin with, patients classified within Cluster 1, those showing more severe clinical involvement, reported significantly higher levels of hopelessness. This finding is consistent with previous studies by Wang et al. (2020) and Fahland et al. (2012), who identified hopelessness as a key mediating factor between CP and emotional decline, particularly in individuals with long-standing, untreated conditions. According to these authors, a negative outlook on the future tends to reinforce avoidance behaviors and social withdrawal, increasing the likelihood of maladaptive responses to pain.

With regard to emotion regulation, results indicated greater levels of dysregulation among patients within the more clinically impaired profile. In this context, Reynolds et al. (2018) highlighted emotional dysregulation as a central mechanism in the worsening of persistent pain in a complex interrelationship with other variables. Heightened emotional reactivity to physical symptoms may not only amplify distress but also interfere with functional recovery. In line with these findings, Attali et al. (2023) observed that difficulties in managing negative emotional states, such as anger or sadness, were closely linked to increased pain intensity and more frequent disruptions in activities of daily life. In this way, mood is a subject of interest for further research. One should bear in mind that emotional dysregulation has been identified as a pivotal mechanism in the exacerbation of CP, intricately interwoven with other contributing factors (Dolgin, 2024). Heightened emotional reactivity to physical sensations can intensify distress and impede functional recovery, potentially explaining the observed link between clinical impairment and emotion regulation deficits (Dunne & Law, 2024).

Beyond the previously discussed aspects, delayed diagnosis and prolonged disease progression often contribute to patients’ perceptions of being a burden to those around them. These clinical factors appear to increase physical dependency and amplify psychosocial strain, particularly as autonomy declines (Tait et al., 2021). Moreover, this subjective burden has been associated with poorer outcomes in terms of perceived pain intensity (Kowal et al., 2012).

Regarding MiL, patients in Cluster 2, those presenting with less severe clinical conditions, reported higher levels of purpose and an active search for meaning. This finding aligns with the study by Boring et al. (2022), who concluded that a strong sense of meaning, especially when grounded in alignment with personal values, serves as a protective buffer against the stress associated with CP and promotes more adaptive coping. Additionally, maintaining a clear sense of purpose not only supports emotional resilience but also enhances engagement in rehabilitation processes and adherence to self-care practices (Almeida et al., 2020).

Reflective functioning also showed a tendency toward greater impairment among individuals in the group with more severe clinical involvement. In this regard, Wiech (2016) shown that the capacity to mentalize one’s own and others’ mental states plays a fundamental role in pain perception, as it allows for a reinterpretation of bodily sensations in ways that may be less debilitating. These findings are consistent with earlier work elaborated by Petrovic and Ingvar (2002), who found that cognitive mechanisms can actively modulate the individual experience of pain, reducing both its intensity and its unpleasantness. Conversely, deficits in reflective capacity may intensify catastrophic thinking and limit patients’ ability to engage in supportive and meaningful interpersonal relationships.

Differences also emerged in relation to coping strategies. While distraction may serve as a helpful short-term mechanism, sustained reliance on this strategy has been linked to avoidant coping patterns and poor functional adjustment among subjects with CP (Van Ryckeghem et al., 2018). However, no differences were found across groups, suggesting the equal use of this coping strategy. The presence of this behavioral pattern may suggest that, in contexts of elevated clinical burden, patients are more likely to adopt passive strategies that limit the assimilation of their condition, offering only temporary relief while ultimately reinforcing emotional distress (Volders et al., 2015), and the results suggest that passive coping tendencies may be common across clinical profiles, regardless of overall symptom severity or impairment.

The results suggest that emotional dysregulation and cognitively oriented coping strategies (mental control, information seeking) are more sensitive to clinical differences between more vulnerable CP patients than affective–expressive or spiritual coping styles. In this regard, several studies have suggested that when patients predominantly use passive strategies, such as avoidance or resignation, their overall emotional and functional adjustment tends to worsen over time. For instance, Van Ryckeghem et al. (2018), McCracken and Samuel (2007), and Philips (1987) pointed out that although distraction might provide temporary relief by shifting attention away from pain, a habitual reliance on such strategies can lead to greater pain interference and worsening psychological outcomes in the long term. In a similar direction, Meulders (2019), Gatchel et al. (2016), and Volders et al. (2015) emphasized that constant avoidance, particularly through repetitive distraction, may end up reinforcing emotional distress, while limiting the management of pain as it progresses.

In contrast, and according to the literature, patients who adopt active coping strategies, such as cognitive restructuring, seeking social support, and problem-solving, tend to engage more effectively with the illness, adjust to its physical and emotional consequences, and report a higher QoL (Montero-Cuadrado et al., 2025; Peres & Lucchetti, 2010). Drawing from both strands of evidence, the literature suggests that interventions promoting active illness management are associated with meaningful improvements in functional capacity and reductions in emotional distress. These findings are consistent with the coping model proposed by Lazarus and Folkman (1987), which posits that approaches such as problem-solving and cognitive reframing foster better emotional and functional adaptation under conditions of chronic stress, such as persistent pain.

Building on the preceding discussion, the results obtained in the present study contribute to a more nuanced understanding of CP. They reveal two clearly differentiated patient profiles based on clinical variables related to diagnostic timelines, disease progression, and QoL. The identification of these profiles provides meaningful insight into how extended diagnostic delays and a prolonged illness duration are linked to heightened psychological vulnerability. This vulnerability becomes visible through elevated levels of hopelessness, perceived burden, and emotional dysregulation, as well as through the prevalent use of maladaptive coping strategies. In contrast, patients with shorter diagnostic delays and lower functional impact appear to possess protective psychological traits, including a more developed sense of purpose and greater reflective capacity.

Despite its strengths, the present study is not without limitations. As is common in research based on self-administered questionnaires, there is a potential for self-report bias, as participants’ responses may be influenced by social desirability. This study is limited by the use of bivariate analyses without multivariate controls, which restricts the ability to account for potential confounding factors such as age, gender, socioeconomic status, or comorbidities. Additionally, the cross-sectional design prevents any causal interpretation. Additionally, the representativeness of the sample may limit the generalizability of the findings, given the substantial predominance of women over men in the participant group. Lastly, women were overrepresented, reflecting both their higher prevalence of CP and greater participation in pain-related research studies.

Future research would benefit from including more balanced and diverse samples, particularly in terms of gender distribution, to capture the variability of psychological responses across different clinical and demographic contexts. Moreover, longitudinal designs could help assess the stability of coping profiles over time and their predictive value for psychological well-being and treatment adherence.

In sum, the findings here highlight the clinical relevance of early diagnosis and prompt intervention, particularly when aimed at mitigating the negative consequences of CP on patients’ QoL and minimizing its long-term psychological impact. Addressing these issues at earlier stages may improve not only clinical outcomes but also the broader psychosocial adjustment of individuals living with persistent pain.

## 5. Conclusions

Through an exploratory approach, distinct patient profiles were identified based on key clinical variables, namely, disease progression, diagnostic delay, and QoL, underscoring the considerable heterogeneity present among individuals living with CP. The more clinically impaired group also showed significantly higher levels of psychological vulnerability, including greater hopelessness, emotional dysregulation, and reduced MiL. These findings highlight the importance of addressing both clinical and psychological dimensions in CP care. Emotion regulation difficulties and existential distress may serve as key indicators of risk, suggesting the need for integrated, person-centered interventions that go beyond symptom management to support psychological resilience.

## Figures and Tables

**Table 1:** Group descriptives across cluster groups.

	Group	N	Mean	SD	SE	CV
PIL-Sense of Life	1	145	21.566	7.356	0.611	0.341
	2	106	27.698	8.101	0.787	0.292
PIL-Propose	1	145	18.476	5.171	0.429	0.280
	2	106	20.981	5.368	0.521	0.256
RFQ8—Uncertainty	1	145	0.840	0.745	0.062	0.887
	2	106	0.575	0.533	0.052	0.926
RFQ8—Certainty	1	145	0.867	0.804	0.067	0.928
	2	106	1.006	0.720	0.070	0.715
Perceived Burdensomeness	1	145	21.041	10.776	0.895	0.512
	2	106	13.349	8.799	0.855	0.659
Frustrated Belongingness	1	145	41.683	7.656	0.636	0.184
	2	106	40.368	6.502	0.632	0.161
BHS—Hopelessness	1	145	10.641	5.279	0.438	0.496
	2	106	6.934	5.229	0.508	0.754
DERS—Dysregulation	1	145	86.648	20.015	1.662	0.231
	2	106	72.792	21.953	2.132	0.302
Prayer	1	145	12.448	7.027	0.584	0.564
	2	106	11.453	6.638	0.645	0.580
Catharsis	1	145	12.510	4.981	0.414	0.398
	2	106	11.792	4.724	0.459	0.401
Distraction	1	144	17.611	5.625	0.469	0.319
	2	105	17.438	6.256	0.610	0.359
Mental Control	1	145	12.724	5.320	0.442	0.418
	2	106	11.075	5.343	0.519	0.482
Self-Affirmation	1	145	17.800	4.638	0.385	0.261
	2	106	17.953	5.298	0.515	0.295
Information Seeking	1	145	19.076	4.637	0.385	0.243
	2	106	16.915	5.011	0.487	0.296

Notes: Decimals have been expressed using points. PIL—Purpose In Life, RFQ—Reflective Function Questionnaire, BHS—Beck Hopelessness Scale, DERS—Difficulties in Emotion Dysregulation Scale. CV—Coefficient Variation, SD—Standard Deviation, SE—Standard Error.

**Table 2:** Independent samples *t*-test.

						95% CI for Cohen’s d	
	*T*	df	*p*	Cohen’s d	SE Cohen’s d	Lower	Upper
PIL-Sense of Life	−6.250	249	<0.001	−0.799	0.136	−1.058	−0.538
PIL-Propose	−3.731	249	<0.001	−0.477	0.131	−0.730	−0.222
RFQ8—Uncertainty	3.120	249	0.002	0.399	0.130	0.145	0.651
RFQ8—Certainty	−1.420	249	0.157	−0.181	0.128	−0.432	0.070
Perceived Burdensomeness	6.025	249	<0.001	0.770	0.136	0.510	1.029
Frustrated Belongingness	1.431	249	0.154	0.183	0.128	−0.068	0.434
BHS—Hopelessness	5.518	249	<0.001	0.705	0.134	0.446	0.962
DERS—Dysregulation	5.199	249	<0.001	0.664	0.134	0.407	0.921
Prayer	1.135	249	0.258	0.145	0.128	−0.106	0.396
Catharsis	1.153	249	0.250	0.147	0.128	−0.104	0.398
Distraction	0.229	247	0.819	0.029	0.128	−0.222	0.281
Mental Control	2.421	249	0.016	0.309	0.129	0.057	0.561
Self-Affirmation	−0.243	249	0.808	-0.031	0.128	−0.281	0.219
Information Seeking	3.524	249	<0.001	0.450	0.130	0.196	0.703

Notes: Decimals have been expressed using points. PIL—Purpose In Life, RFQ—Reflective Function Questionnaire, BHS—Beck Hopelessness Scale, DERS—Difficulties in Emotion Dysregulation Scale.

## Data Availability

On request to the corresponding author.
